# Making connections: exploring the centrality of posttraumatic stress symptoms and covariates after a terrorist attack

**DOI:** 10.1080/20008198.2017.1333387

**Published:** 2017-06-02

**Authors:** Marianne Skogbrott Birkeland, Trond Heir

**Affiliations:** ^a^ Norwegian Centre for Violence and Traumatic Stress Studies, Oslo, Norway; ^b^ Institute of Clinical Medicine, Faculty of Medicine, University of Oslo, Oslo, Norway

**Keywords:** PTSD, network analysis, psychopathology, terrorism, aetiology

## Abstract

**Background**: Posttraumatic stress symptoms are interconnected. Knowledge about which symptoms of posttraumatic stress are more strongly interconnected or central than others may have implications for the targeting of clinical interventions. Exploring whether symptoms of posttraumatic stress may be differentially related to covariates can contribute to our knowledge on how posttraumatic stress symptoms arise and are maintained.

**Objective**: This study aimed to identify the most central symptoms of posttraumatic stress and their interconnections, and to explore how covariates such as exposure, sex, neuroticism, and social support are related to the network of symptoms of posttraumatic stress.

**Method**: This study used survey data from ministerial employees collected approximately 10 months after the 2011 Oslo bombing that targeted the governmental quarters (*n* = 190). We conducted network analyses using Gaussian graphical models and the lasso regularization.

**Results**: The network analysis revealed reliably strong connections between intrusive thoughts and nightmares, feeling easily startled and overly alert, and between feeling detached and emotionally numb. The most central symptom in the symptom network was feeling emotionally numb. The covariates were generally not found to have high centrality in the symptom network. An exception was that being female was connected to a high physiological reactivity to reminders of the trauma.

**Conclusions**: Ten months after a workplace terror attack emotional numbness appears to be of high centrality in the symptom network of posttraumatic stress. Fear circuitry and dysphoric symptoms may constitute two functional entities in chronic posttraumatic stress. Clinical interventions targeting numbness may be beneficial in the treatment of posttraumatic stress, at least after workplace terrorism.

## 1. Introduction

Exposure to a traumatic event often results in psychological reactions such as re-experiencing the event, avoiding reminders of trauma, and increased anxiety and emotional arousal. Researchers have explored how factors of posttraumatic symptoms may influence each other longitudinally (Marshall, Schell, Glynn, & Shetty, 2006; Pietrzak et al., 2014; Schell, Marshall, & Jaycox, 2004; Solberg, Birkeland, Blix, Hansen, & Heir, 2016; Solomon, Horesh, & Ein-Dor, 2009). Common findings among these studies are that intrusive and hyperarousal symptoms predict further symptom development. However, symptom clusters may have effects with shorter time spans than are possible to detect in such longitudinal studies, which have months or years between measurement waves. Furthermore, other symptom constellations or single symptoms may be central to the development or maintenance of posttraumatic stress symptoms.

The network perspective constitutes an alternative conceptualization of posttraumatic symptomatology that can offer a way to overcome these limitations. According to this perspective, disorders can be understood as networks of interacting symptoms more than as underlying disease entities that produce the symptoms (Borsboom & Cramer, 2013; Hofmann, Curtiss, & McNally, 2016; McNally, 2016; Nuijten, 2016). For example, a person who experiences a traumatic event may develop certain symptoms (e.g. intrusive memories) that activate other symptoms (e.g. insomnia), which in turn activate other symptoms (e.g. difficulty concentrating). These latter symptoms may have connections back to the first symptoms (intrusive memories), creating feedback loops of symptoms that maintain each other. By identifying which symptoms are more strongly connected to or more central than others, we can gain information about possible powerful targets for clinical interventions (Fried et al., 2016).

A small number of studies have explored networks of posttraumatic stress symptoms (Armour, Fried, Deserno, Tsai, & Pietrzak, 2017; Bryant et al., 2016; McNally, 2016; McNally et al., 2015). There were some similarities across the findings of these studies. For example, in all studies strong associations were found between hypervigilance and an exaggerated startle response, and between nightmares and flashbacks. However, different symptoms were found to be the most central in the networks; for example hypervigilance and future foreshortening (McNally et al., 2015), feeling detached (McNally, 2016), negative trauma-related emotions, flashbacks, detachment, and physiological cue reactivity (Armour et al., 2017), or intrusions and physiological reactivity (Bryant et al., 2016). The contradictory results may be due to differences in the trauma populations that were studied. The first study examined people who were grieving a lost child and dealing with practical difficulties following an earthquake (McNally et al., 2015), the second examined people with a history of childhood sexual abuse (McNally, 2016), the sample in the third study had experienced a wide range of traumas (Armour et al., 2017), and the fourth study included hospitalized patients with traumatic injuries (Bryant et al., 2016). These experiences are quite different, and they may not necessarily elicit the same network of symptoms. More studies of various trauma-affected populations are needed to determine whether networks of posttraumatic stress symptoms differ according to the type of traumatic event. Furthermore, the networks characteristics may differ in immediate phases compared to in chronic phases (Bryant, 2003).

In line with the studies that have identified factor or groups of posttraumatic stress symptoms (e.g. Armour, Műllerová, & Elhai, 2016), the network of posttraumatic stress symptoms may contain subnetworks with symptoms that are more tightly connected with each other than with other symptoms. These communities or clusters of symptoms may function as relatively independent modules of a network (Fortunato, 2010). Examining grouping of posttraumatic stress symptoms from a network perspective may shed light on whether and how subsets of symptoms can constitute functional entities that might correspond to specialized functional modules.

Factors outside the posttraumatic stress symptoms defined by the diagnosis manuals may also contribute to the dynamics of the posttraumatic stress response. Among the well-known risk factors or covariates of posttraumatic stress are high severity of exposure (Santiago et al., 2013), being female (Olff, Langeland, Draijer, & Gersons, 2007), low levels of social support (Charuvastra & Cloitre, 2008; Ozer, Best, Lipsey, & Weiss, 2003), and high neuroticism (Ormel et al., 2013). Despite the numerous studies that have investigated these factors as predictors of posttraumatic stress, few have examined why or how they lead to the development of posttraumatic stress symptomatology. Examining the relationships between these covariates and the network of posttraumatic stress symptoms may provide new information about the processes by which these factors exert their negative influence.

In this study, we extend previous network studies on posttraumatic stress in two ways: (1) we sampled a population of individuals who had experienced a bomb explosion at their workplace. Thus, all of these people experienced the same event, and they were measured at approximately the same time after the event; (2) we included variables in the network that can be considered risk factors for or covariates of posttraumatic stress symptoms, such as severity of exposure, sex, social support, and neuroticism, to determine whether they were differentially associated with the posttraumatic stress symptoms.

The aim of our study was threefold. The first aim was to explore which symptoms are more centrally located in a network of posttraumatic stress symptoms 10 months after a terrorist attack. Secondly, we aimed to identify sub-networks of symptoms that are more tightly connected with each other. Thirdly, we aimed to explore the role that specific covariates such as severity of exposure, sex, social support, and neuroticism play in the networks of posttraumatic stress symptoms.

## 2. Method

### 2.1. Participants

The participants were ministerial employees present during the 2011 Oslo bombing attack, which was a terrorist attack directed toward the Norwegian government ministries. On 22 July 2011, a car bomb exploded in the executive governmental quarter. The blast damaged governmental buildings, killed eight people, and injured 209 people. The data for this study were collected 10 months after the attack, in April/May 2012. All of the employees in 14 of the 17 Norwegian ministries were invited to participate in a research project titled ‘Mental health and work environment factors in the aftermath of the Oslo terrorist attack July 22nd, 2011’ (Hansen et al., in press). When the bomb exploded, 342 people were at work in the targeted governmental buildings. They were mainly bureaucrats and administrative leaders. As they were inside the targeted buildings when the bomb went off, they were all directly exposed to a death threat, and fulfil criterion A for PTSD both according to DSM-IV and DSM-5.

Of these 342 persons, 207 responded to the survey (60.5%). Among these, 17 individuals did not complete the measure of posttraumatic stress and were excluded from the first network analysis (*n* = 190). Another two people did not complete the measures of the covariates and were excluded from the network analysis with covariates (*n* = 188).

All of the participants were informed about the purpose and content of the study, and they were given the opportunity to withdraw from the study. The study was approved by the Regional Ethics Committee in Norway.

### 2.2. Measures

Posttraumatic stress was assessed with the Norwegian version of the PCL-S (Hem, Hussain, Wentzel-Larsen, & Heir, 2012). The PCL-S is a 17-item self-administered questionnaire that assesses PTSD symptom severity (per DSM-IV), and the items were specifically linked to the bomb explosion. The respondents were asked to rate the extent to which they had been bothered by PTSD symptoms over the last four weeks on a five-point scale that ranged from ‘Not at all’ (1) to ‘Extremely’ (5). The symptom ratings were summarized to reflect a total score. Cronbach’s alpha for the total PCL-S was .94.

We constructed a severity of exposure value by assessing the participants’ number of exposure types. Respondents were asked whether they had: (a) witnessed people who were dead or dying; (b) witnessed people who were seriously injured; and (c) whether they themselves had been physically injured. The response for each item was coded as 0 or 1 and summarized to create a severity of exposure value that ranged from 0 to 3.

We measured social support with four items from the Crisis Support Scale (CSS) (Joseph, Andrews, Williams, & Yule, 1992). The items ‘Someone willing to listen’, ‘Able to talk about thoughts and feelings’, ‘Sympathy and support from others’, and ‘Practical help’ were categorized as positive social support. Because all of the respondents were part of a government district workforce, the last positive social support item, ‘Contact with others in a similar situation’, was omitted. Responses for these items were measured on a seven-point scale that ranged from ‘Never’ (1) to ‘Always’ (7), and item scores were averaged to reflect a total score. Cronbach’s alpha for this scale was .89.

Neuroticism at T1 was measured using the subscale for this construct from the 44-item Big Five Inventory (John, Donahue, & Kentle, 1991). Examples of items include ‘I see myself as someone who is depressed’ and ‘I see myself as someone who is relaxed and handles stress well’. The respondents rated these items on a five-point scale that ranged from ‘disagree strongly’ (1) to ‘agree strongly’ (5). After reversing the scores for positively framed items, item scores were averaged to yield an overall neuroticism score, with high scores representing high neuroticism. Cronbach’s alpha for this scale was .83.

### 2.3. Statistical analyses

#### 2.3.1. Network estimation

We used the R package qgraph (Epskamp, Cramer, Waldorp, Schmittmann, & Borsboom, 2012) to compute several networks. Each of the 17 DSM-IV PTSD symptoms was represented by a node in the network, and the strength of the association between the symptoms (nodes) was represented by an edge between the nodes. Following guidelines presented by Epskamp, Borsboom, and Fried (2017) and Epskamp and Fried (2016), we computed networks by employing the ‘least absolute shrinkage and selection operator’ (graphical LASSO, glasso; Friedman, Hastie, & Tibshirani, 2008), implemented in qgraph. This procedure identifies only the relevant edges and accurately discovers the underlying network structures. The tuning parameter (gamma) for the glasso estimation was 0.5. The resulting estimates of edges can be interpreted as the correlation between two symptoms after controlling for all other symptoms in the network (partialling out common variance between nodes). For all of the variables, we used the cor auto function in the qgraph package, which automatically computes the appropriate correlations for the variable types.

#### 2.3.2. Centrality analysis

We computed each symptom’s centrality in the network. The higher the centrality of a symptom, the more strongly that symptom is connected to other symptoms in the network (Borsboom & Cramer, 2013; Epskamp et al., 2017). Three common types of centrality were assessed: strength, closeness, and betweenness. The strength of a node indicates the mean magnitude of the correlations of each edge linked to the node. It provides a measure of how strongly a node is directly connected to other nodes in the network. The closeness of a node takes the inverse of the sum of all shortest paths between a node and all other nodes in the network, investigating how strongly a node is indirectly connected to other nodes in the network. The betweenness of a node indicates the number of times the node lies on the shortest path between two other nodes. It can be interpreted as how central the node is in connecting other nodes.

#### 2.3.3. Robustness of networks

To ensure that the estimated networks were robust enough for interpretation, we followed the recommendations by Epskamp et al. (2017) and performed robustness analyses using the R-package bootnet. Bootstrapping procedures were used to examine two robustness-related issues: (1) the robustness of the edge weights; and (2) the robustness of the measures of node centrality. We assessed the variability of edge weights and centrality by estimating confidence intervals (CI) in which 95% of the cases contain the true value of the parameter (bootstrapped samples = 1000). The network stability can be quantified by using the correlation stability (CS) coefficient. This measure quantifies the maximum proportion of cases that can be dropped to retain, with 95% certainty, a correlation with the original centrality higher than (by default) 0.7. Based on a simulation study, Epskamp, Borsboom, and Fried suggest that to interpret centrality differences the CS-coefficient should not be below .25 and preferably above .50 (Epskamp et al., 2017).

#### 2.3.4. Community detection

To investigate whether the nodes cluster together, we implemented a modularity based community detecting algorithm. We used the spin-glass algorithm, which tests for communities in the network whereby the number and weighted strength of edges within a cluster exceed the number and weighted strength of edges between nodes in another cluster (Reichardt & Bornholdt, 2006). We applied the spin-glass community function of the R package igraph over the glasso network (weights = null, vertex = null, parupdate = false, gamma = 0.5, start temperature = 1, stop temperature = 0.01, cooling factor = 0.99, spins = 17) (Csardi & Nepusz, 2006).

#### 2.3.5. Visualization

The network structure is visualized by using the Fruchterman–Reingold algorithm (Fruchterman & Reingold, 1991), which places nodes with stronger and/or more connections closer together, and nodes with weaker connections are placed more peripherally in the network (Epskamp et al., 2012). Positive edges are printed in green, negative edges are printed in red, and thicker and more saturated edges indicate stronger connections. We set the maximum edge value to 0.46, the strongest edge identified across our networks. This makes it possible to compare saturation and thickness across networks. To enhance the interpretability of the graphs, we used a minimum value of 0.03 in all networks. We have shown symptoms from a given symptom cluster in the five-factor model (Elhai & Palmieri, 2011) in the same colour. Also, the covariates are shown in the same colour.

## 3. Results

### 3.1. Characteristics of the sample

As can be seen in Table 1, all respondents were present in the buildings near where the bomb exploded, and a majority (66%) witnessed seriously injured people. Twenty-four percent of the sample reported PTSD symptoms that meet the criteria for probable PTSD according to the flowchart method (Hansen, Nissen, & Heir, 2013). In line with the specifications of DSM-IV, this procedure requires one positive score in cluster B, three in cluster C, and two in cluster D (Hem et al., 2012). Furthermore, 22% of the respondents reported a sum of PTSD symptoms over the PCL cut point at 45, and 50% reported a sum of PTSD symptoms over the PCL cut point at 30 (VA National Center for PTSD, 2012).

**Table 1.** Characteristics of the sample.

	*N* ≈ 207
Age (years) M ± SD	44.7 (11.9)
Gender (female %)	61
Education (low/mid/high %)	10/29/62
	
Posttraumatic stress (PCL-17) M ± SD	34.11 (14.96)
Percentage fulfilling criteria for PTSD	24
	
Did you witness dead/dying people? (yes %)	34
Did you witness seriously injured people? (yes %)	66
Were you injured? (yes %)	25
Was a colleague injured? (yes %)	53
Did a colleague of yours die? (yes %)	19
Office damage? (yes %)	66

Table 2 shows the distributions of the symptom scores. Among the most endorsed symptoms were intrusions (*R1*), difficulty concentrating (*DA3*), being irritable/angry (*DA2*), and easily startled (*AA2*).

**Table 2.** Distribution of the symptom scores (range 1–5); means, standard deviations and skewness (*n* = 190).

	Mean	SD	Skewness
R1: intrusive thoughts	2.52	1.21	0.37
R2: nightmares	1.66	1.00	1.57
R3: reliving trauma	1.72	0.97	1.26
R4: emotional cue reactivity	2.25	1.23	0.78
R5: physiological cue activity	1.97	1.21	1.08
A1: avoidance of thoughts	2.11	1.21	0.90
A2: avoidance of reminders	1.78	1.07	1.17
N1: trauma-related amnesia	1.83	1.12	1.27
N2: loss of interest	1.87	1.18	1.25
N3: feeling detached	1.92	1.20	1.12
N4: feeling numb	1.50	0.96	1.94
N5: hopelessness	1.55	0.98	1.82
DA1: difficulty sleeping	2.13	1.34	0.88
DA2: irritable/angry	2.31	1.26	0.54
DA3: difficulty concentrating	2.49	1.33	0.51
AA1: overly alert	2.19	1.25	0.66
AA2: easily startled	2.30	1.31	0.63

### 3.2. Network of posttraumatic stress symptoms

**Figure 1.**
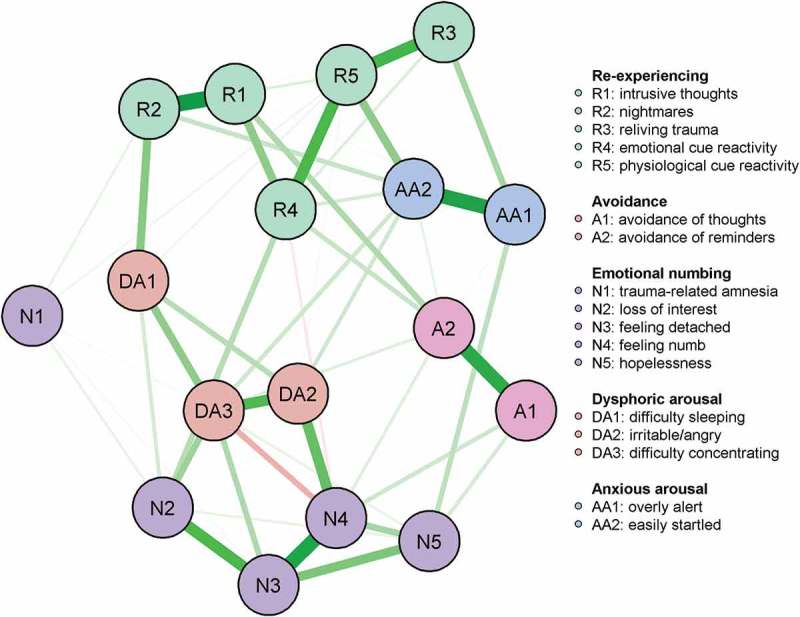
Network of the symptoms of posttraumatic stress 10 months after experiencing a terrorist attack.

Figure 1 shows the glasso network structure for the 17 DSM-IV PTSD symptoms. Of the possible 136 edges between symptoms, 70 were retained and estimated in the glasso estimation. Most of the symptoms were positively correlated with each other. The 95% CI around the edge weights can be viewed in the supplementary materials (see Figure S1 in Supplemental data). The generally large bootstrapped CIs suggest that interpreting the order of most edges in the network should be done carefully. Many of the bootstrapped CIs overlap each other, suggesting that many edge weights do not significantly differ from one another. However, several of the edge weights were reliably stronger than most of the others (see Figure S2). Among these were the edge weights between intrusive thoughts (*R1*) and nightmares (*R2*), feeling easily startled (*AA1)* and overly alert (*AA2*), and between feeling detached (*N3*) and feeling emotionally numb (*N4*).

We also computed measures of node centrality (betweenness, closeness, and strength) for the glasso network, and computed correlation stability (CS) coefficients. The CS coefficients indicated that betweenness (CS(cor = 0.7) = 0.05) and closeness (CS(cor = 0.7) = 0.21) were not stable under subsetting cases. The measure of strength (CS(cor = 0.7) = 0.28) performed better. As the CS coefficients should exceed 0.25 to be interpretable, we concluded that the order of the node strength was interpretable with some care, whereas the orders of betweenness and closeness were not (see also Figure S3).

**Figure 2.**
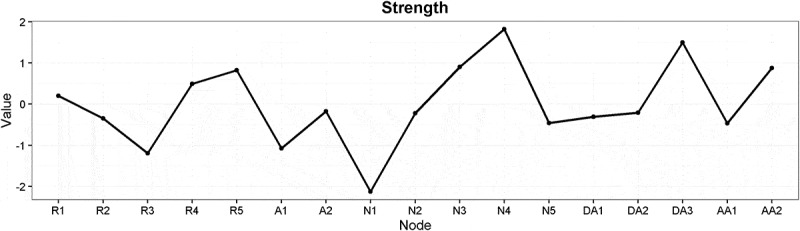
Estimates of node strength for the symptoms of posttraumatic stress.

Figure 2 presents centrality plot of strength for the nodes in the glasso network. Among the nodes with the strongest measure of node strength were feeling emotionally numb (*N4*), concentration difficulties (*DA3*), feeling detached from other people (*N3*), physiological cue reactivity (*R5*), and feeling easily startled (*AA2*). However, most values of node strengths could not be shown to significantly differ from each other (see Figure S4 in Supplemental data). An exception was feeling emotionally numb (*N4*) which was significantly higher in strength than several of the other nodes.

The spin-glass algorithm detected two functionally distinct communities of nodes in the glasso network. One community comprised all the re-experiencing, avoidance, and anxious arousal symptoms. A second community comprised all the emotional numbing and dysphoric arousal symptoms.

### 3.3. Network of posttraumatic stress symptoms with covariates

**Figure 3.**
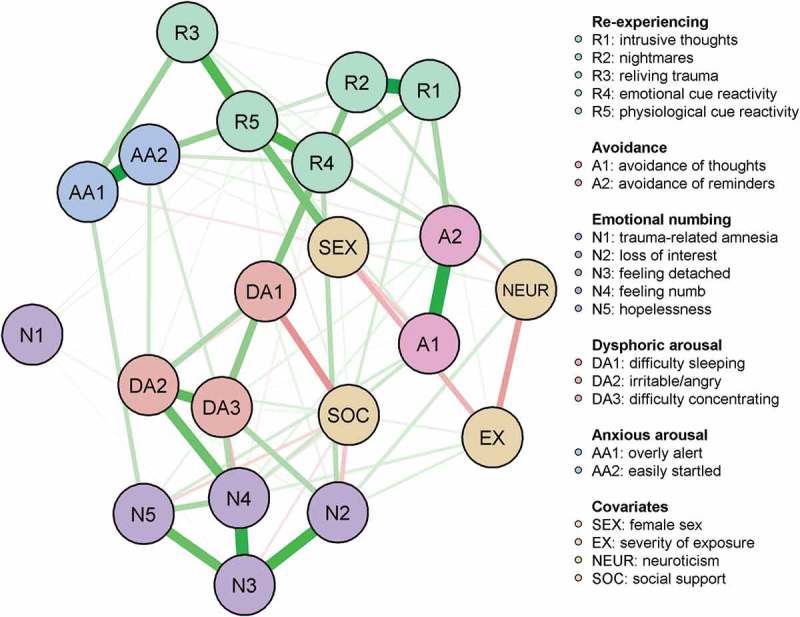
Network of the symptoms of posttraumatic stress with covariates 10 months after experiencing a terrorist attack.

We also computed a glasso network of the 17 DSM-IV PTSD symptoms together with covariates of posttraumatic stress, including exposure, sex, social support, and neuroticism. Of the possible 210 edges between nodes, 112 were estimated. This network is presented in Figure 3 (robustness of the edge weights can be examined in Figure S5). Again, we found generally large bootstrapped CIs, and many edge weights overlapped with each other. Most of the edge weights involving the correlates could not be identified to be significantly stronger than other edge weights (see Figure S6). However, the connection between being female (*sex*) and higher physiological cue reactivity (*R5*) was significantly stronger than many of the other edge weights. Furthermore, being female (*sex*) was also connected to lower levels of avoiding thoughts and feelings (*A1*). High severity of exposure (*ex*) was connected to feeling emotionally numb (*N4*) and loss of interest in previously enjoyed activities (*N2*). Low levels of social support (*soc*) were connected to more sleeping problems (*DA1*) and loss of interest in previously enjoyed activities (*N2*). Neuroticism (*neur*) was connected to nightmares (*R1*) and loss of interest in previously enjoyed activities (*N2*).

**Figure 4.**
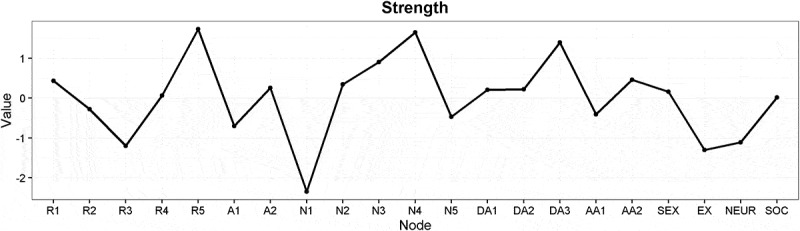
Estimates of node strength for the symptoms of posttraumatic stress and covariates.

Again, the CS coefficients indicated that betweenness (CS(cor = 0.7) = 0.13) and closeness (CS(cor = 0.7) = 0.21) were not stable under subsetting cases, strength (CS(cor = 0.7) = 0.28) performed better. We concluded that the order of the node strength was interpretable with some care, whereas the orders of betweenness and closeness were not (see also Figure S7). The centrality estimates are shown in Figure 4. Generally, we found similar results as in the symptom network. Most values of node strengths could not be shown to significantly differ from each other (see Figure S8). An exception is that neuroticism (*neur*) had significantly lower strength than feeling emotionally numb (*N4*).

## 4. Discussion

The present study explored the network of posttraumatic stress symptoms after exposure to a terrorist attack. The most endorsed symptoms included intrusions, having difficulty concentrating, being irritable or angry, and feeling easily startled. Numbing was not among the most endorsed symptoms of posttraumatic stress in this sample, yet it was the most central symptom in the network. Our results resemble the findings from a study of the posttraumatic stress symptoms network 12 months after being injured (Bryant et al., 2016). In other studies, other symptoms were found to be more central, such as concentration difficulties and future foreshortening (McNally et al., 2015), or negative trauma-related emotions and flashbacks (Armour et al., 2017). These differences may be due to the inherent differences in exposure and traumatic experiences across samples as well as differences in timing of the study. Furthermore, these studies used different versions of the DSM. By utilizing DSM-IV, we had no opportunity to explore the potential roles of the symptoms of negative beliefs, blame of self or others, or reckless behaviour, which is included in DSM-5. However, even though some the symptom descriptions are reworded, many of them are basically the same. With some care, it is possible to compare the results across the studies.

Consistent with previous studies (Armour et al., 2017; Bryant et al., 2016; McNally, 2016; McNally et al., 2015), we found several pairs of symptoms with strong connectivity. For example, hypervigilance and feeling easily startled were highly connected, which may reflect that these symptoms have a common aetiology, or that they may constitute a mutually reinforcing feedback loop (McNally et al., 2015). Intrusions and traumatic dreams also were strongly connected, which may indicate a loop in which repeated intrusive waking recollections of the traumatic event make traumatic dreams more probable, and traumatic dreams, in turn, may make traumatic memories even more intrusive.

As in the previous network studies (Armour et al., 2017; Bryant et al., 2016; McNally, 2016; McNally et al., 2015), we also found that feeling detached was strongly connected to both feeling emotionally numb and loss of interest in previously enjoyed activities. The importance of numbing is consistent with studies suggesting a link between PTSD and difficulty regulating negative emotions (Shepherd & Wild, 2014). Emotional numbing may be viewed as a form of emotion regulation in which the emotional response is modulated by disengaging from rather than engaging with emotions. This involves automatic processes that diminish experiencing negative, and especially positive affects after trauma (Litz, Orsillo, Kaloupek, & Weathers, 2000). Accordingly, when disengaging from one’s emotions, there is also the danger of disengaging emotionally from other people (feeling distant) and positive activities (loss of interest in previously enjoyed activities).

Trauma-related amnesia was not found to have a central role in the symptom network. This is one of the most consistent findings across the network studies (Armour et al., 2017; McNally et al., 2015), and it is also consistent with findings from other studies (Geraerts & McNally, 2008; Rubin, Berntsen, & Bohni, 2008). This suggests that trauma-related amnesia may have limited utility when examining posttraumatic stress symptoms and should perhaps not be regarded a core symptom of posttraumatic stress disorder. On the contrary, posttraumatic stress symptomatology has been found to be closely related to vivid memories of the traumatic event (Porter & Peace, 2007).

We found evidence for two potential sub-networks within the posttraumatic symptomatology; one comprising the re-experiencing, avoidance, and anxious arousal symptoms, and another comprising emotional numbing and dysphoric arousal symptoms. Again, our findings resemble findings from Bryant and colleagues’ study, where it was found that 12 months after exposure to physical trauma, fear circuitry and dysphoric symptoms emerged as core elements of the chronic stress response (Bryant et al., 2016).

In general, our results indicated that the covariates had low centrality in the network of posttraumatic stress symptoms. However, some results are worth mentioning. For example, being female was connected with physiological reactivity to reminders of the trauma. Stress hormones enhance consolidation in episodic memory, particularly for emotionally arousing information. Sex hormones may impact the effects of stress on emotional learning and memory (Merz & Wolf, 2017), which again have implications for physiological reactivity to reminders of trauma.

That low social support was connected to sleeping difficulties is consistent with studies reporting an association between low levels of social support and sleep disturbances (Åkerstedt et al., 2002; Brummett et al., 2006). Low levels of social support after exposure to a traumatic event may lead to sleep disturbances because individuals are deprived of emotional and instrumental support, which may lead to rumination and higher activation, which hinder sleep.

We found no evidence for connectivity between neuroticism and the posttraumatic stress symptoms. One explanation for this may be that neuroticism may act as a confounding factor that is connected with all the symptoms (Engelhard, Hout, & Lommen, 2009; Ormel et al., 2013). Another possibility is that what we term "neuroticism" may not reflect an underlying latent separate entity at all, but rather a network of affective, cognitive, and behavioural components that interact (Cramer et al., 2012). This is similar to what we have measured with the measure of posttraumatic stress symptoms. Accordingly, neuroticism may not have an informative role in the aetiology of posttraumatic stress symptomatology.

In addition, severity of exposure was weakly connected to the posttraumatic stress symptomatology. The generally low centrality of exposure severity and the other covariates considered here may be due to the timing of the study. These data were collected 10 months after the incident, when some individuals may have regained their resilience or recovered from the transient phase of posttraumatic reactions. However for others, the system of PTSD symptoms may have reached the tipping point to move from a state of strong resilience to a state of high connectivity between symptoms (Hofmann et al., 2016). Thus, for these latter individuals, the system of symptoms may have reached an equilibrium state of reciprocally reinforcing symptoms – which is often termed chronic PTSD. Either way, the covariates may no longer have an active function in maintaining the symptoms.

One of the strengths of this study is our use of network analysis, which may add to our understanding of posttraumatic processes and how symptoms are maintained over time. We measured symptoms of posttraumatic stress in individuals who had experienced the same event 10 months before measurement. We also assessed the robustness of the estimates.

Among the limitations of this study is the use of self-report cross-sectional data, which limits our ability to identify direct causal influences among symptoms. The low sample size may contribute to the moderate stability of the estimates of the edge weights and centrality measures. Unfortunately, we have no data on possible ongoing or prior treatment, which makes it difficult to determine to what extent the results from this sample are representative for clinical samples or community populations. Another limitation is the broad range of posttraumatic stress symptoms among our participants (rather than examining only people with clinical PTSD). Thus, caution is required in drawing conclusions for patients in clinical settings based on these data. The results may be more relevant in a general public context, in which people react in different degrees to traumatic events such as terrorist attacks.

## 5. Conclusions

We found that numbing was a highly central symptom of posttraumatic stress 10 months after a workplace terrorist attack. Thus, interventions that successfully reduce numbing may initiate beneficial effects that limit other symptoms, thereby speeding up recovery. However, when comparing our findings with those from similar studies, it becomes apparent that the posttraumatic stress symptoms that are most central can vary according to the type of trauma that individuals experience. Nevertheless, many of the connections between the symptoms seem to be similar across type of exposure. Taken together, this may be interpreted as indicating that, even though the symptoms of posttraumatic stress may arise under different circumstances, the mutually reinforcing processes that constitute PTSD may be similar.

Further research should use longitudinal time series data (experience sampling method) perhaps also recorded in acute phases, which would allow researchers to identify relationships and connections that occur over short time spans. This may provide information on temporal dynamics that can contribute to studying the tipping points at which people suddenly shift from a healthy state to a chronic state of PTSD. Including multiple indicators, such as observational data on physiological activation, sleep, or cognitive tasks, can reveal how automatic processes that cannot be assessed by self-reports are involved in maintaining the symptoms of posttraumatic stress. In addition, by testing interventions that target specific symptoms, one can test whether the symptoms are changeable and whether reducing them helps to slow down the network and increase recovery from posttraumatic stress symptoms.

## Supplementary Material

Supplementary material
