# Supplementary material for: Making connections: exploring the centrality of posttraumatic stress symptoms and covariates after a terrorist attack
Source: Eur J Psychotraumatol. 2017 Jun 2;8(sup3):1333387. doi: 10.1080/20008198.2017.1333387 (PMC5632769; doi:10.1080/20008198.2017.1333387)
Supplement: Supplementary material [file ZEPT_A_1333387_SM5807.pdf]

## Supplemental figure Legends

Figure S1.

Bootstrapped confidence intervals of estimated edge weights for the network of posttraumatic stress symptoms (Figure 1 in the main manuscript). The red line indicates the sample values and the gray area the 95% CIs.

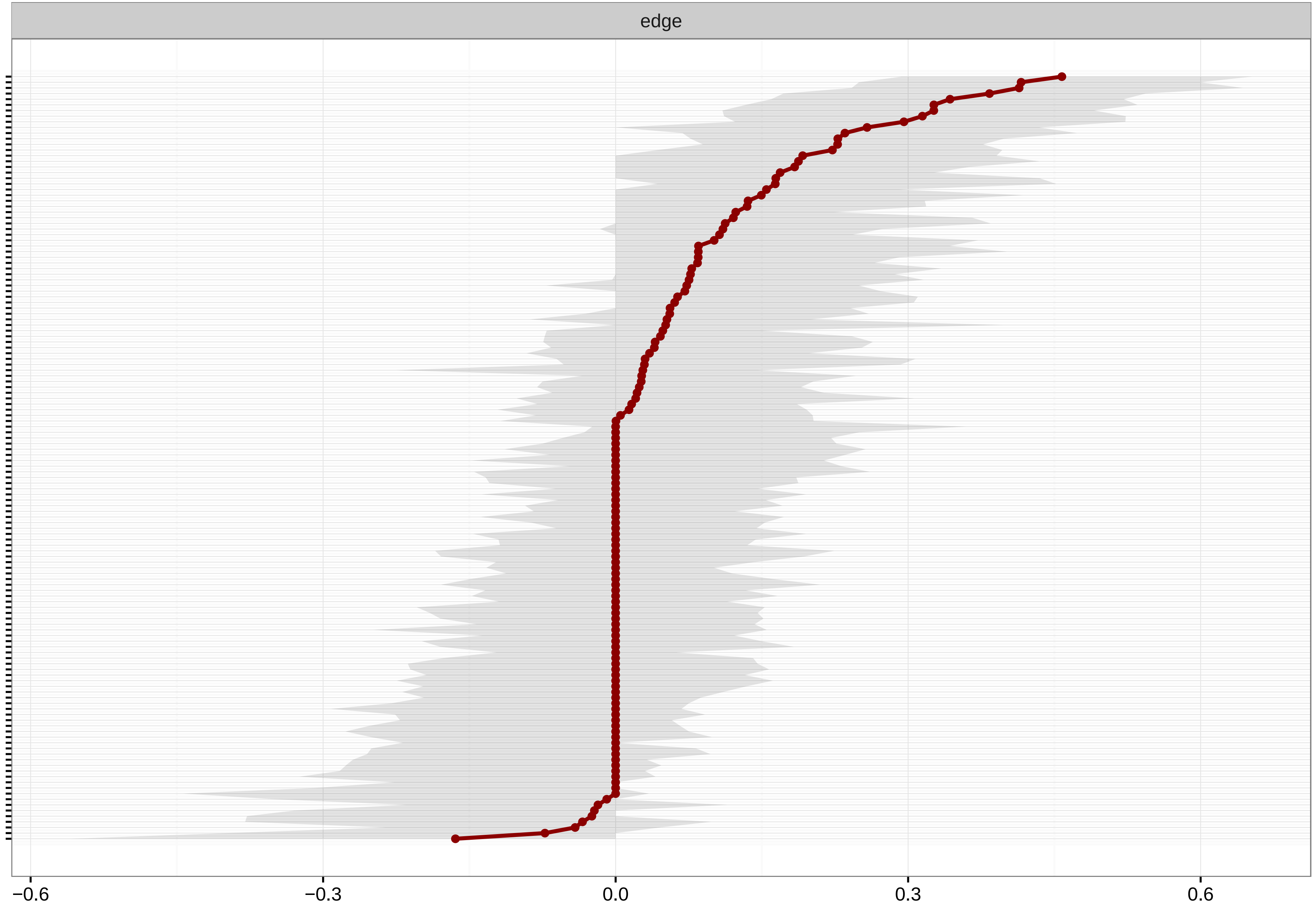

edge
−0.6
−0.3
0.0
0.3
0.6

Figure S2.

Bootstrapped difference tests (α = 0.05) between edge-weights that were non-zero in the network of posttraumatic stress symptoms (gray boxes indicate edges that do not differ significantly from one-another and black boxes represent edges that do differ significantly from one-another).

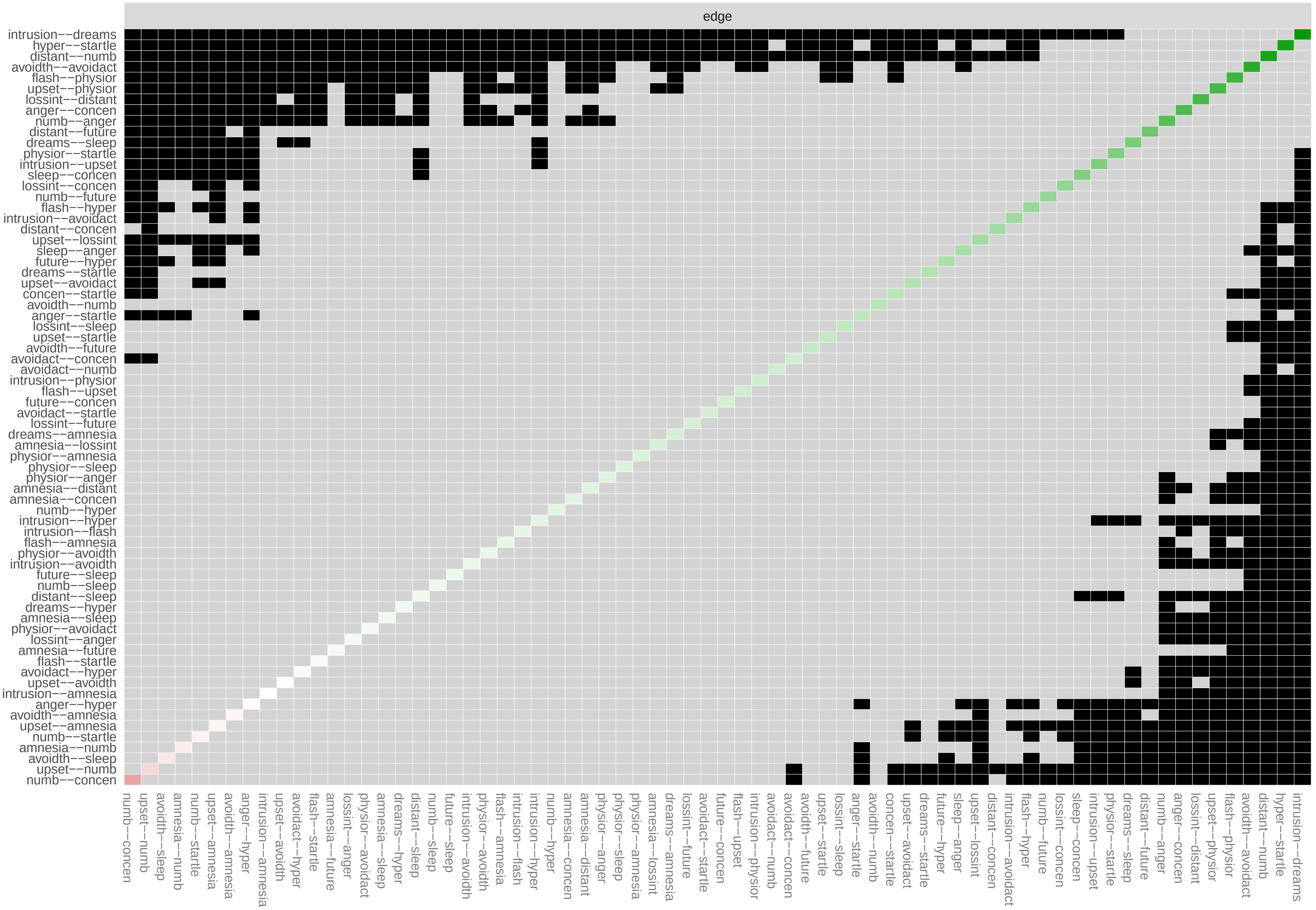

edge
intrusion−−dreams
hyper−−startle
distant−−numb
avoidth−−avoidact
flash−−physior
upset−−physior
lossint−−distant
anger−−concen
numb−−anger
distant−−future
dreams−−sleep
physior−−startle
intrusion−−upset
sleep−−concen
lossint−−concen
numb−−future
flash−−hyper
intrusion−−avoidact
distant−−concen
upset−−lossint
sleep−−anger
future−−hyper
dreams−−startle
upset−−avoidact
concen−−startle
avoidth−−numb
anger−−startle
lossint−−sleep
upset−−startle
avoidth−−future
avoidact−−concen
avoidact−−numb
intrusion−−physior
flash−−upset
future−−concen
avoidact−−startle
lossint−−future
dreams−−amnesia
amnesia−−lossint
physior−−amnesia
physior−−sleep
physior−−anger
amnesia−−distant
amnesia−−concen
numb−−hyper
intrusion−−hyper
intrusion−−flash
flash−−amnesia
physior−−avoidth
intrusion−−avoidth
future−−sleep
numb−−sleep
distant−−sleep
dreams−−hyper
amnesia−−sleep
physior−−avoidact
lossint−−anger
amnesia−−future
flash−−startle
avoidact−−hyper
upset−−avoidth
intrusion−−amnesia
anger−−hyper
avoidth−−amnesia
upset−−amnesia
numb−−startle
amnesia−−numb
avoidth−−sleep
upset−−numb
numb−−concen
numb−−concen
upset−−numb
avoidth−−sleep
amnesia−−numb
numb−−startle
upset−−amnesia
avoidth−−amnesia
anger−−hyper
intrusion−−amnesia
upset−−avoidth
avoidact−−hyper
flash−−startle
amnesia−−future
lossint−−anger
physior−−avoidact
amnesia−−sleep
dreams−−hyper
distant−−sleep
numb−−sleep
future−−sleep
intrusion−−avoidth
physior−−avoidth
flash−−amnesia
intrusion−−flash
intrusion−−hyper
numb−−hyper
amnesia−−concen
amnesia−−distant
physior−−anger
physior−−sleep
physior−−amnesia
amnesia−−lossint
dreams−−amnesia
lossint−−future
avoidact−−startle
future−−concen
flash−−upset
intrusion−−physior
avoidact−−numb
avoidact−−concen
avoidth−−future
upset−−startle
lossint−−sleep
anger−−startle
avoidth−−numb
concen−−startle
upset−−avoidact
dreams−−startle
future−−hyper
sleep−−anger
upset−−lossint
distant−−concen
intrusion−−avoidact
flash−−hyper
numb−−future
lossint−−concen
sleep−−concen
intrusion−−upset
physior−−startle
dreams−−sleep
distant−−future
numb−−anger
anger−−concen
lossint−−distant
upset−−physior
flash−−physior
avoidth−−avoidact
distant−−numb
hyper−−startle
intrusion−−dreams

Figure S3.

Average correlations between centrality indices of networks of posttraumatic stress symptoms sampled with persons dropped and the original sample. Lines indicate the means and areas indicate the range from the 2.5th quantile to the 97.5th quantile.

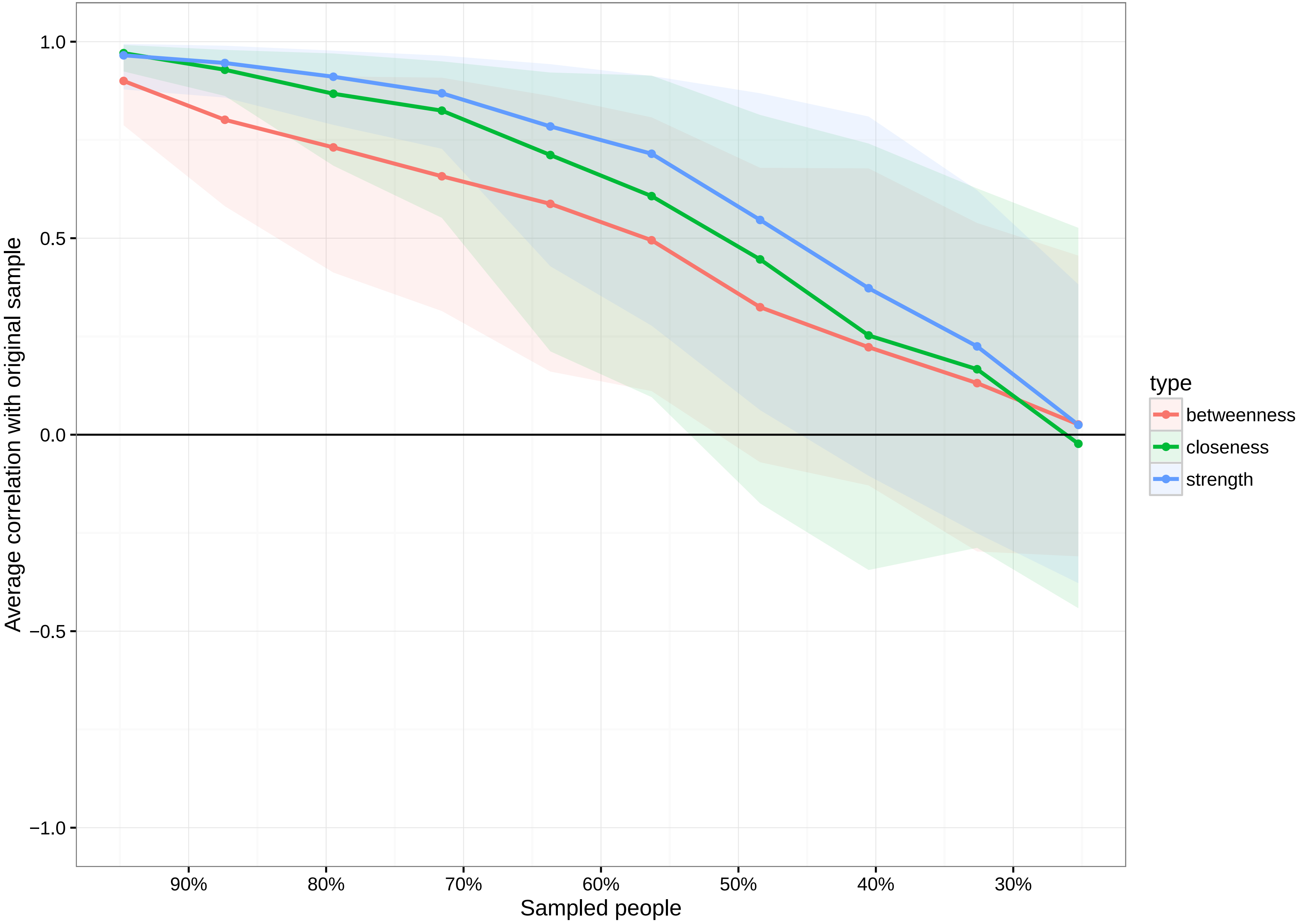

1.0
0.5
0.0
−0.5
−1.0
Average correlation with original sample
90%
80%
70%
60%
50%
40%
30%
Sampled people
type
betweenness
closeness
strength

Figure S4.

Bootstrapped difference tests ($\alpha = 0.05$) between node strengths that were non-zero in the network of posttraumatic stress symptoms (gray boxes indicate nodes that do not differ significantly from one-another and black boxes represent nodes that do differ significantly from one-another).

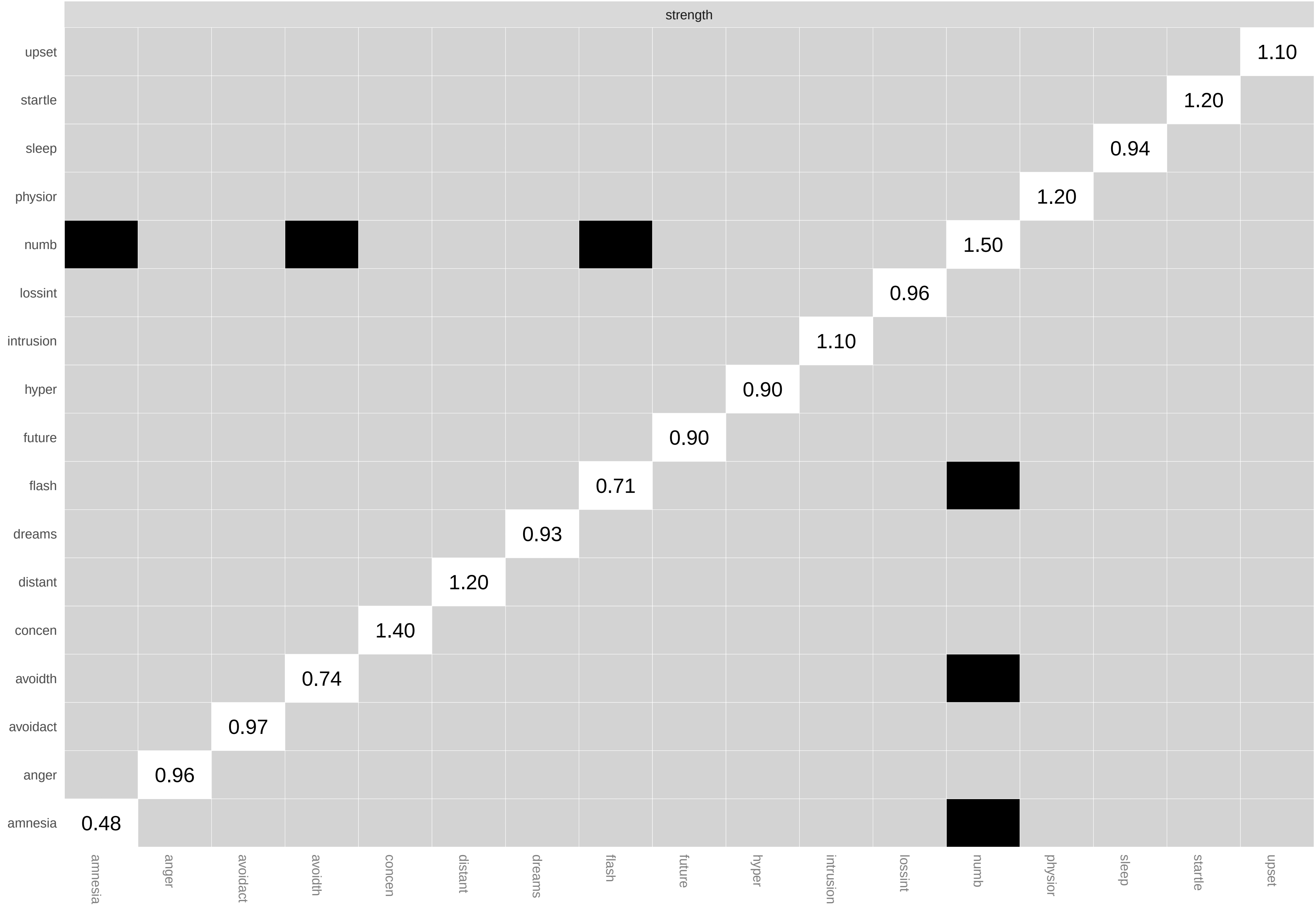
strength
upset
startle
sleep
physior
numb
lossint
intrusion
hyper
future
flash
dreams
distant
concen
avoidth
avoidact
anger
amnesia
1.10
1.20
0.94
1.20
1.50
0.96
1.10
0.90
0.90
0.71
0.93
1.20
1.40
0.74
0.97
0.96
0.48
amnesia
anger
avoidact
avoidth
concen
distant
dreams
flash
future
hyper
intrusion
lossint
numb
physior
sleep
startle
upset

Figure S5.

Bootstrapped confidence intervals of estimated edge weights for the network of the symptoms of posttraumatic stress along with four risk factors (Figure 3 in the main manuscript). The red line indicates the sample values and the gray area the 95% CIs.

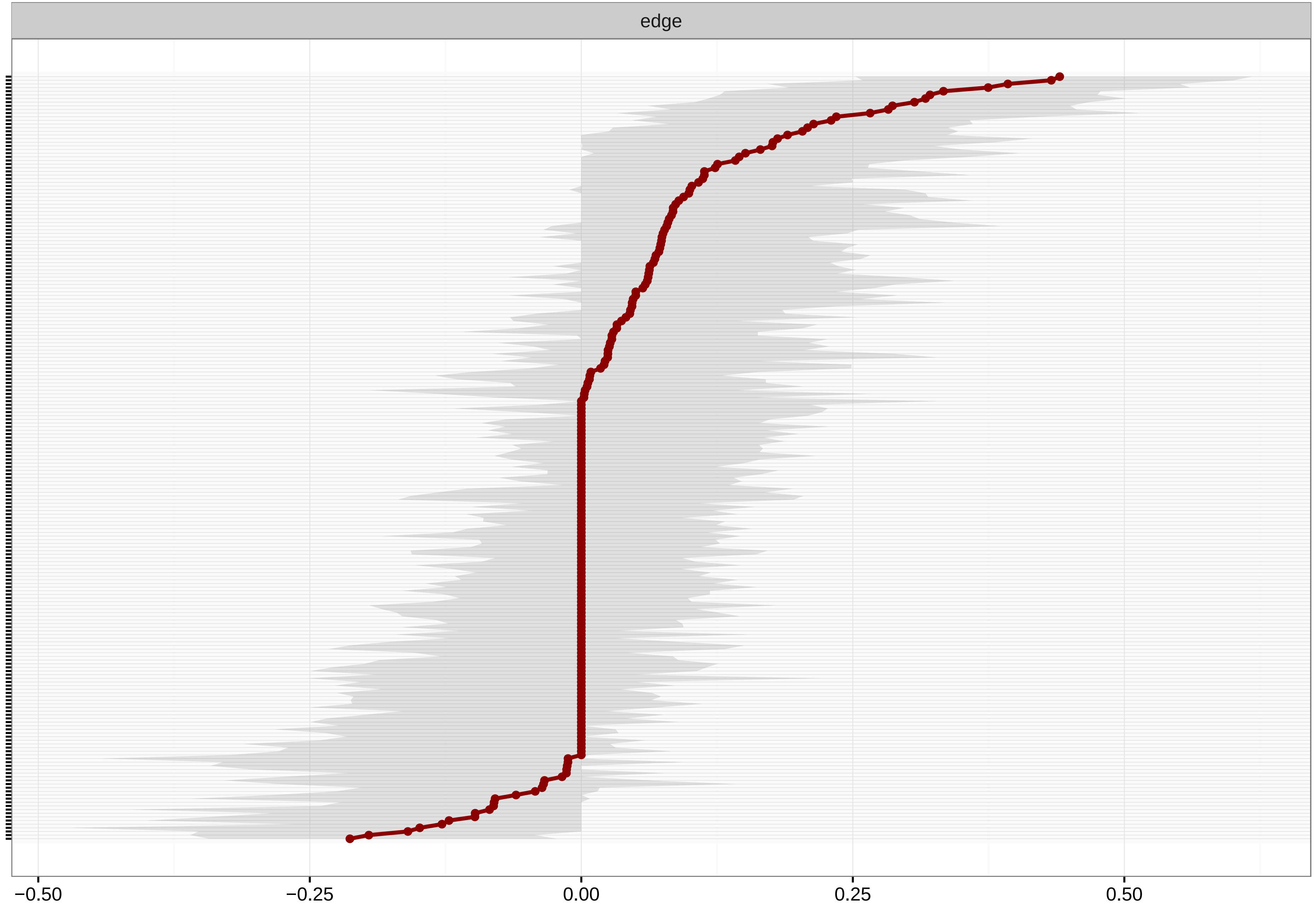

edge
−0.50
−0.25
0.00
0.25
0.50

Figure S6.

Bootstrapped difference tests (α = 0.05) between edge-weights that were non-zero in the network of posttraumatic stress symptoms with covariates (gray boxes indicate edges that do not differ significantly from one-another and black boxes represent edges that do differ significantly from one-another).

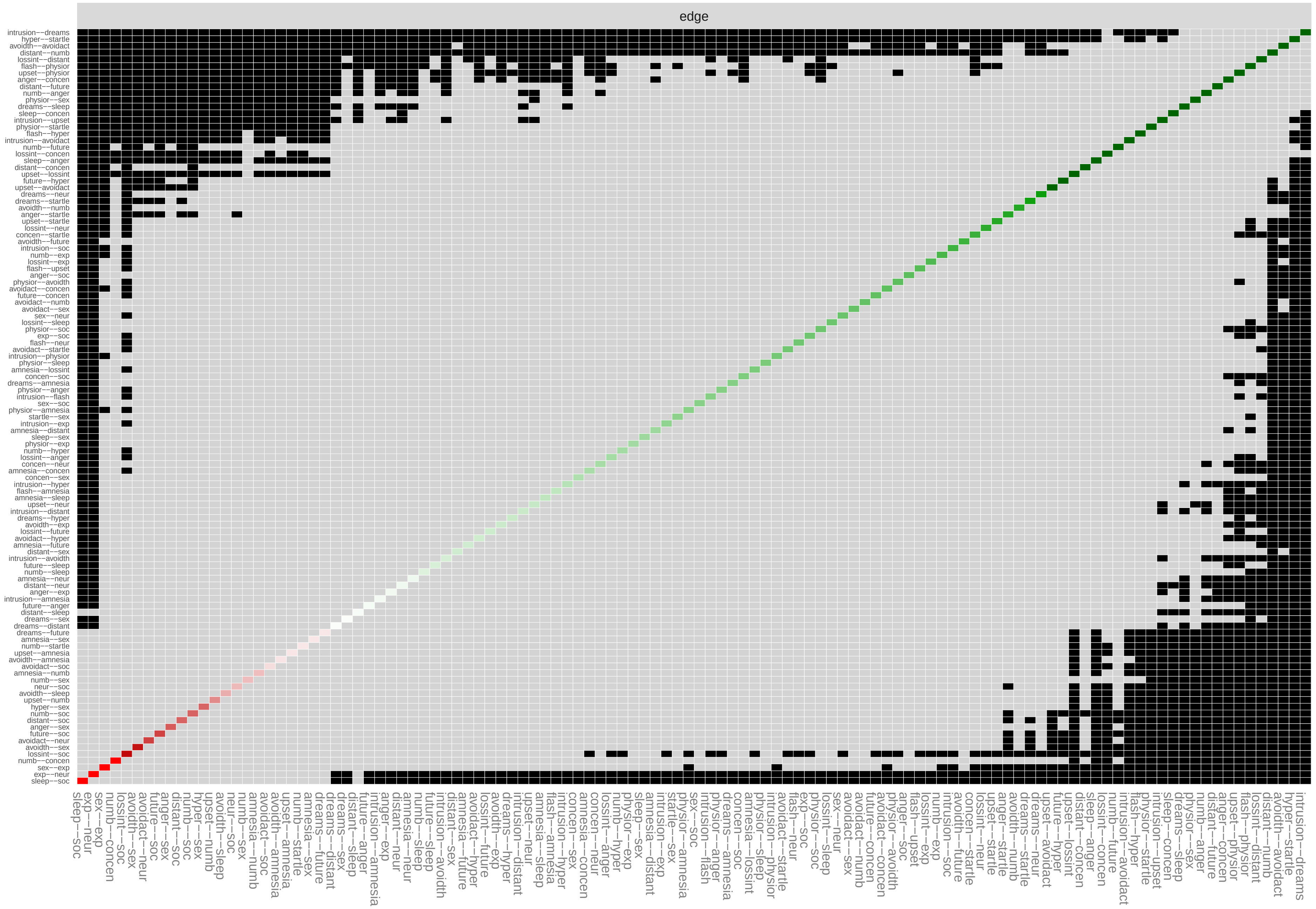
edge
intrusion--dreams
hyper--startle
avoidth--avoidact
distant--numb
lossint--distant
flash--physior
upset--physior
anger--concen
distant--future
numb--anger
physior--sex
dreams--sleep
sleep--concen
intrusion--upset
physior--startle
flash--hyper
intrusion--avoidact
numb--future
lossint--concen
sleep--anger
distant--concen
upset--lossint
future--hyper
upset--avoidact
dreams--neur
dreams--startle
avoidth--numb
anger--startle
upset--startle
lossint--neur
concen--startle
avoidth--future
intrusion--soc
numb--exp
lossint--exp
flash--upset
anger--soc
physior--avoidth
avoidact--concen
future--concen
avoidact--numb
avoidact--sex
sex--neur
lossint--sleep
physior--soc
exp--soc
flash--neur
avoidact--startle
intrusion--physior
physior--sleep
amnesia--lossint
concen--soc
dreams--amnesia
physior--anger
intrusion--flash
sex--soc
physior--amnesia
startle--sex
intrusion--exp
amnesia--distant
sleep--sex
physior--exp
numb--hyper
lossint--anger
concen--neur
amnesia--concen
concen--sex
intrusion--hyper
flash--amnesia
amnesia--sleep
upset--neur
intrusion--distant
dreams--hyper
avoidth--exp
lossint--future
avoidact--hyper
amnesia--future
distant--sex
intrusion--avoidth
future--sleep
numb--sleep
amnesia--neur
distant--neur
anger--exp
intrusion--amnesia
future--anger
distant--sleep
dreams--sex
dreams--distant
dreams--future
amnesia--sex
numb--startle
upset--amnesia
avoidth--amnesia
avoidact--soc
amnesia--numb
numb--sex
neur--soc
avoidth--sleep
upset--numb
hyper--sex
numb--soc
distant--soc
anger--sex
future--soc
avoidact--neur
avoidth--sex
lossint--soc
numb--concen
sex--exp
exp--neur
sleep--soc
sleep--soc
exp--neur
sex--exp
numb--concen
lossint--soc
avoidth--sex
avoidact--neur
future--soc
anger--sex
distant--soc
numb--soc
hyper--sex
upset--numb
avoidth--sleep
neur--soc
numb--sex
amnesia--numb
avoidact--soc
avoidth--amnesia
upset--amnesia
numb--startle
amnesia--sex
dreams--future
dreams--distant
dreams--sex
distant--sleep
future--anger
intrusion--amnesia
anger--exp
distant--neur
amnesia--neur
numb--sleep
future--sleep
intrusion--avoidth
distant--sex
amnesia--future
avoidact--hyper
lossint--future
avoidth--exp
dreams--hyper
intrusion--distant
upset--neur
amnesia--sleep
flash--amnesia
intrusion--hyper
concen--sex
amnesia--concen
concen--neur
lossint--anger
numb--hyper
physior--exp
sleep--sex
amnesia--distant
intrusion--exp
startle--sex
physior--amnesia
sex--soc
intrusion--flash
physior--anger
dreams--amnesia
concen--soc
amnesia--lossint
physior--sleep
intrusion--physior
avoidact--startle
flash--neur
exp--soc
physior--soc
lossint--sleep
sex--neur
avoidact--sex
avoidact--numb
future--concen
avoidact--concen
physior--avoidth
anger--soc
flash--upset
lossint--exp
numb--exp
intrusion--soc
avoidth--future
concen--startle
lossint--neur
upset--startle
anger--startle
avoidth--numb
dreams--startle
dreams--neur
upset--avoidact
future--hyper
upset--lossint
distant--concen
sleep--anger
lossint--concen
numb--future
intrusion--avoidact
flash--hyper
physior--startle
intrusion--upset
sleep--concen
dreams--sleep
physior--sex
numb--anger
distant--future
anger--concen
upset--physior
flash--physior
lossint--distant
distant--numb
avoidth--avoidact
hyper--startle
intrusion--dreams

Figure S7.

Average correlations between centrality indices of networks of posttraumatic stress symptoms with covariates sampled with persons dropped and the original sample. Lines indicate the means and areas indicate the range from the 2.5th quantile to the 97.5th quantile.

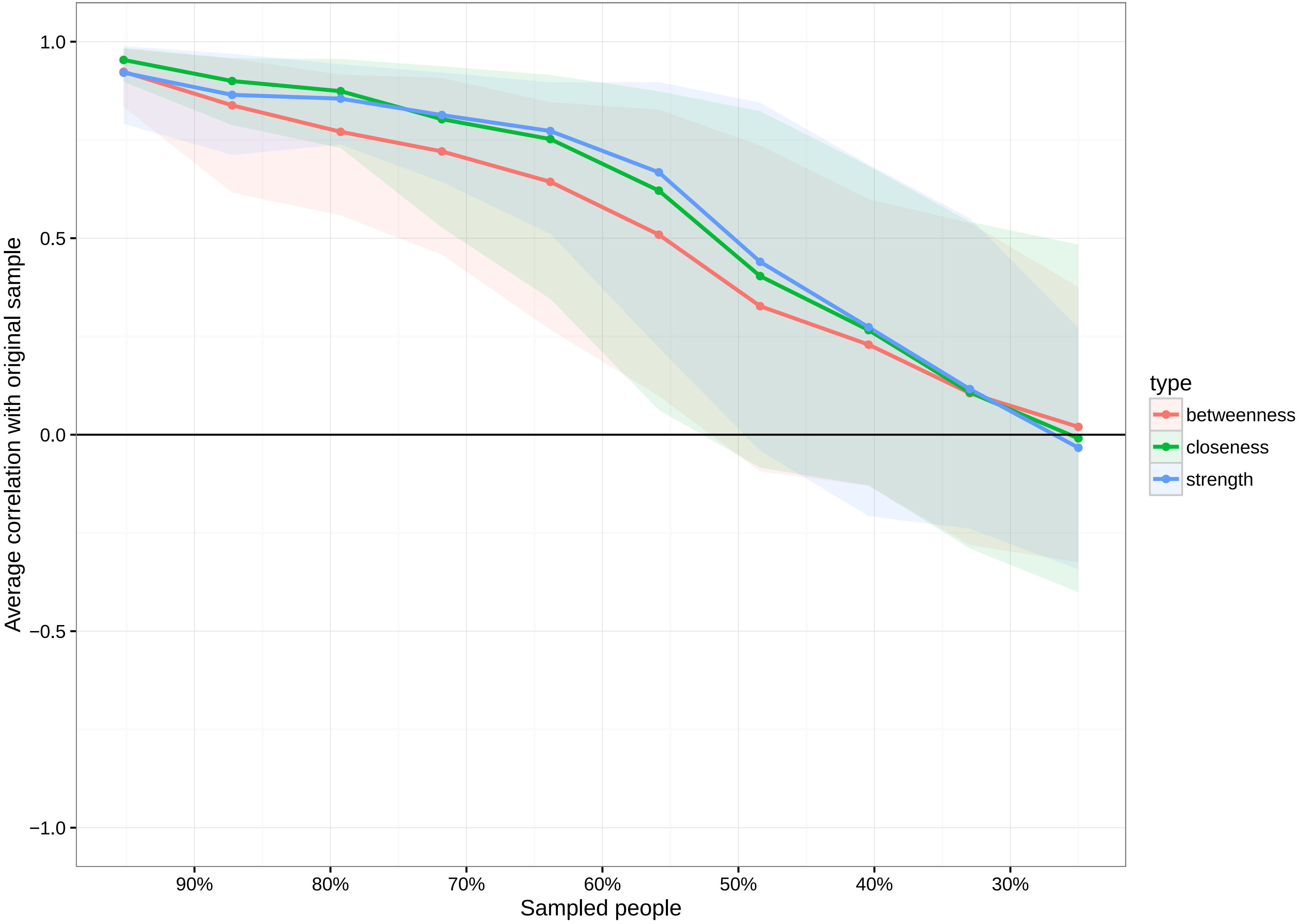

Average correlation with original sample
Sampled people
1.0
0.5
0.0
−0.5
−1.0
90%
80%
70%
60%
50%
40%
30%
type
betweenness
closeness
strength

Figure S8.

Bootstrapped difference tests (α = 0.05) between node strengths that were non-zero in the network of posttraumatic stress symptoms with covariates (gray boxes indicate nodes that do not differ significantly from one-another and black boxes represent nodes that do differ significantly from one-another).

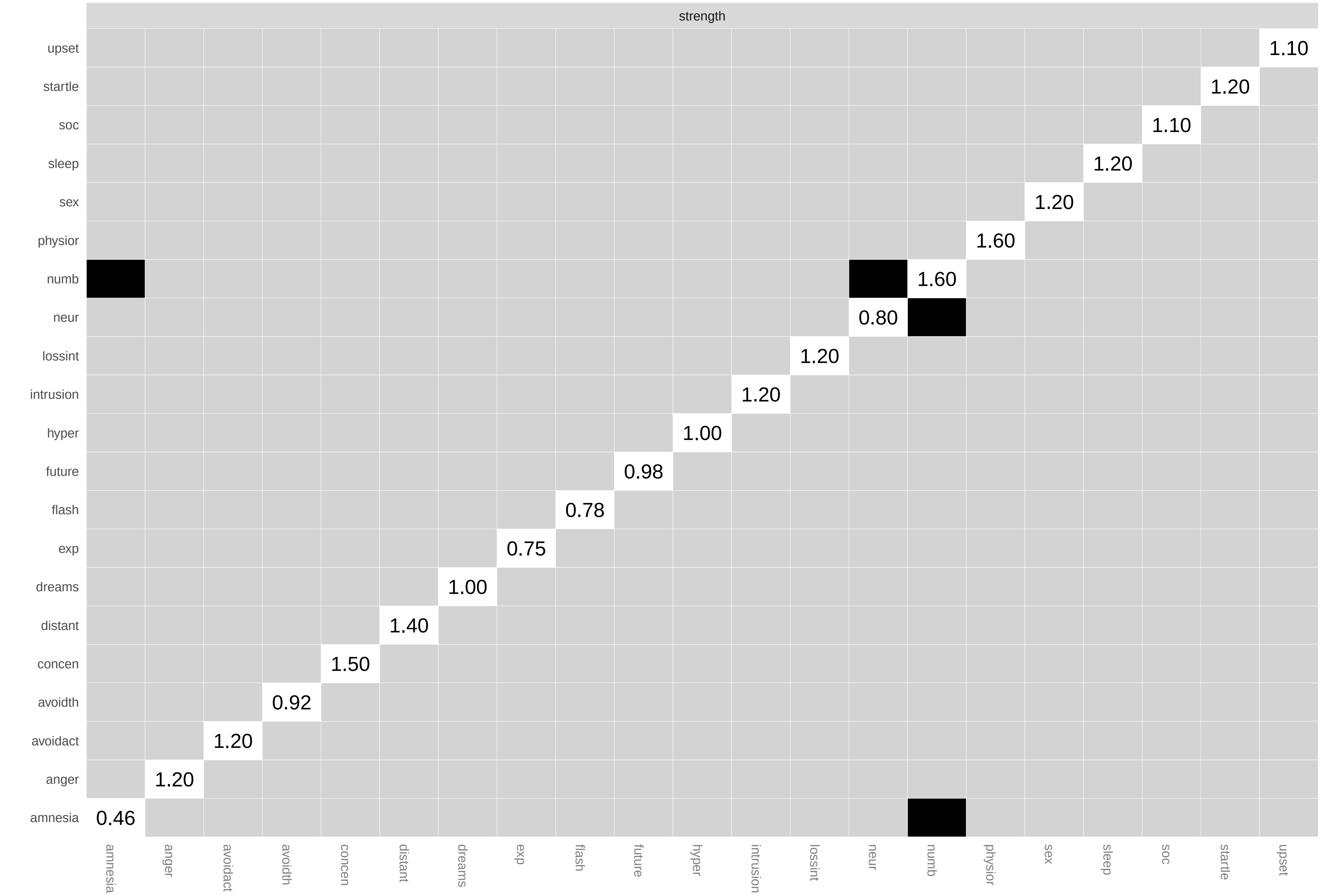

strength
upset
startle
soc
sleep
sex
physior
numb
neur
lossint
intrusion
hyper
future
flash
exp
dreams
distant
concen
avoidth
avoidact
anger
amnesia
1.10
1.20
1.10
1.20
1.20
1.60
1.60
0.80
1.20
1.20
1.00
0.98
0.78
0.75
1.00
1.40
1.50
0.92
1.20
1.20
0.46
amnesia
anger
avoidact
avoidth
concen
distant
dreams
exp
flash
future
hyper
intrusion
lossint
neur
numb
physior
sex
sleep
soc
startle
upset
